# Epidemiology of asthma and associated factors in an urban Pakistani population: adult asthma study-Karachi

**DOI:** 10.1186/s12890-018-0753-y

**Published:** 2018-12-04

**Authors:** Shama Razzaq, Asaad Ahmed Nafees, Unaib Rabbani, Muhammad Irfan, Shahla Naeem, Muhammad Arslan Khan, Zafar Fatmi, Peter Burney

**Affiliations:** 1 Department of Community Health Sciences, Aga Khan University, Stadium Road, P.O. Box-3500, Karachi, 74800 Pakistan; 2 Saudi Board Family Medicine, Ministry of Health, Buraidah, Kingdom of Saudi Arabia; 3 Department of Medicine, Section of Pulmonology, Aga Khan University, Karachi, Pakistan; 4 Respiratory Epidemiology and Public Health, National Heart & Lung Institute, Imperial College London, London, UK; 5 Population Health and Occupational Disease, National Heart and Lung Institute (NHLI), Imperial College London, London, UK; 6 Department of Community Health Sciences, Ziauddin University, Karachi, Pakistan

**Keywords:** Asthma, Adult, Epidemiology, Risk factors, Spirometry, Pakistan

## Abstract

**Background:**

This study was conducted in order to determine the prevalence of asthma and associated risk factors in the adult population of Karachi, Pakistan.

**Methods:**

This multi-stage, cross-sectional survey was conducted from May 2014–August 2015; comprising 1629 adults in 75 randomly selected clusters in Karachi, Pakistan. Definitions included: ‘self-reported asthma’, ‘reversibility in FEV_1_^’^ and ‘respiratory symptoms and reversibility in FEV_1_’.

**Results:**

Prevalence of asthma was 1.8% (self-reported) (95% CI: 1.0–2.6), 11.3% (reversibility in FEV_1_) (95% CI: 9.4–13.3) and 6.6% (symptoms and reversibility in FEV_1_) (95% CI: 5.1–8.1). Asthmatics were more likely to belong to the age group ≥38 years according to ‘reversibility in FEV_1_’ and ‘respiratory symptoms and reversibility in FEV_1_’ (AOR: 1.9, 95% CI: 1.2–3.3) and (AOR: 2.1, 95% CI: 1.1–4.2), respectively. Asthmatics were more likely to report history of allergies (AOR: 1.9, 95% CI: 1.2–2.9) and (AOR: 2.8, 95% CI: 1.7–4.8); and were exposed to environmental tobacco smoke (AOR: 1.6, 95% CI: 1.1–2.5) and (AOR: 1.9, 95% CI: 1.1–3.3) according to ‘reversibility in FEV_1_’ and ‘respiratory symptoms and reversibility in FEV_1_’, respectively. Asthmatics were more likely to report pack years of smoking ≥5 (AOR: 2.3, 95% CI: 1.1–4.7) according to ‘respiratory symptoms and reversibility in FEV_1_’.

**Conclusion:**

This study reports a high prevalence of asthma among Pakistani adults and calls for developing appropriate public health policies for prevention and control of asthma in the country. Further studies should be conducted to determine the national prevalence as well as follow-up studies to identify preventable causes for adult asthma.

**Electronic supplementary material:**

The online version of this article (10.1186/s12890-018-0753-y) contains supplementary material, which is available to authorized users.

## Background

According to available global estimates, around 300 million people currently suffer from asthma, which has become more common in adults in recent decades [1]. One in every 250 deaths worldwide has been attributed to asthma, which was found to rank 26th among causes associated with years of life lost in South Asia [1, 2]. The increase in the prevalence of asthma has been associated with an increase in atopic sensitization, and is paralleled by similar increases in other allergic disorders such as eczema and rhinitis [1]. The rate of asthma increases as communities become more urbanized; with the projected increase in the proportion of the world’s population that is urban from 45 to 59% in 2025, there is likely to be a marked increase in the number of asthmatics worldwide [1].

The known risk factors for developing asthma include a combination of genetic predisposition and environmental exposure to various substances that may provoke allergic reactions or irritate the airways. Such environmental exposures may include house dust mites in bedding, carpets and stuffed furniture, pet dander, pollens and molds, tobacco smoke and chemical irritants [3, 4]. Some additional factors which may exacerbate or cause asthma include work related stress, sedentary lifestyle and indoor air pollutants [5].

Globally, there is a lack of standardized data regarding prevalence of asthma due to variations in the operational definition and assessment techniques [6]. There is a lack of community based studies identifying burden of asthma through spirometry, while most of the studies rely on questionnaires [5, 7, 8]. The question regarding self-reported physician diagnosed asthma has been shown to have high specificity for assessing asthma in several epidemiological surveys worldwide, however, sizeable proportion of asthma may still remain undiagnosed [9]. In order to address this concern, the recent guidelines recommend a combination of questionnaire and spirometry based information as the best method for determining asthma in epidemiological surveys [6, 10].

According to India’s National Family Health Survey, prevalence of adult self-reported asthma was around 1.8% [11]. Asthma prevalence has been found to vary from 0.7 to 11.9% across Asia, but the definitions used to identify asthma also varied extensively [5]. The report on global burden of asthma estimated the prevalence of asthma, in Pakistan to be 4.3% [1]. Employing a robust methodology and using comprehensive set of definitions, this study was conducted in order to determine the prevalence of asthma and associated risk factors in the adult population of Karachi, Pakistan.

## Methods

### Study design and setting

This population based cross-sectional survey was conducted in Karachi, Pakistan, from May 2014 to August 2015. A megacity, and the largest urban center and economic hub of Pakistan, Karachi is estimated to have a population of over 20 million [12]. The city inhabits a mix of various regional and national ethno-linguistic groups while Urdu is the most commonly spoken language [13]. There are an estimated 702 slums in the city harboring 40–61% of the population [13]. The city is administratively divided into six districts; South, East, West, Central, Malir and Korangi; in addition, there are six military cantonments [12].

### Participant recruitment

Multi-stage cluster sampling was used for selection of clusters (primary sampling units) and households (secondary sampling units) in Karachi. Using the sampling frame for clusters or enumeration blocks available from the Pakistan Bureau of Statistics (PBS) (2003), 75 clusters, out of 9400, were randomly selected. Line-listing was carried out within each cluster and 40 households per cluster (out of 250–300) were selected randomly. All eligible participants (adults aged ≥18 years and those living in Karachi in the same household for at least six months) from the selected households were recruited in the study.

The required sample size was calculated to be 1284 participants (using the Open Epi version 2.3) based on prevalence of asthma assessed through spirometry 2% to 3.6% [14], or self-reported 2% to 6.5% [15], keeping bound on the error 1.5% and 2% respectively, confidence level of 95%, a design effect of 2 and inflated by 10% to adjust for non-response and unacceptable quality of spirometry.

### Interviews

Interviews were conducted in the local language by trained field staff using a structured questionnaire that was developed by adapting already published questionnaires for identifying possible risk factors of asthma [16]. It included questions related to the socio-demographic and economic variables (age, gender, socio-economic and educational status); indoor air pollution and ventilation (exposure to environmental tobacco smoke, housing structure, ventilation in house, kitchen characteristics and cooking habits) and other risk factors for asthma (presence of carpet at home, use of air-conditioning or incense and mosquito coils, recent paint or new furniture being brought at home and presence of pets, birds or animals at home). Questions regarding respiratory symptoms and illnesses (cough, sputum, wheeze, shortness of breath and any pre-existing respiratory conditions) including asthma, family history of asthma and other respiratory diseases, smoking habits, and occupation were added from the American Thoracic Society (ATS-DLD-78A) questionnaire, which has been validated in Pakistan [17, 18]. Questionnaire was translated into Urdu and then back translated to English to check the accuracy of translation and pre-tested before use.

### Lung function and anthropometric assessment

Lung function was assessed through spirometry by trained field staff using Vitalograph Alpha spirometer (Vitalograph New Alpha 6000; Vitalograph Ltd., Buckingham, England) according to the ATS guidelines [19, 20]. Participants were explained about the procedure and spirometry was performed in a sitting position with nose clip attached. Forced Vital Capacity (FVC), Forced Expiratory Volume in first second (FEV_1_) and FEV_1_/FVC were recorded in liters. Post-bronchodilator reversibility in FEV_1_ was assessed by administering Salbutamol (200 μg) through a 500-mL spacer device and repeating the test after 15 min. Three manoeuvers were performed and acceptable readings were recorded for both pre and post-bronchodilator response. Participants reporting eye, heart, lung, chest or abdominal surgery in the past 6 months, heart attack in the past 3 months, those with pulse greater than 120 beats per minute, recent respiratory infections (tuberculosis or pneumonia) and those who were pregnant at the time of data collection were excluded from performing spirometry. Anthropometric measurements including height and weight were also taken.

### Definitions

Three distinct definitions for asthma were used; including ‘self-reported, physician-diagnosed asthma’ based on information from the questionnaire; ‘reversibility in FEV_1_’, assessed on presence of post-bronchodilator reversibility ≥200 ml in FEV_1_; ‘respiratory symptom(s) and reversibility in FEV_1_’, based on post-bronchodilator reversibility ≥200 ml in FEV_1_ and one or more respiratory symptoms, or self-reported asthma [10]. ‘Acute cough or phlegm’ was defined as cough or phlegm as much as 4 to 6 times a day in a week and/or first thing in morning and/or at all during the rest of the day or at night. ‘Chronic cough or phlegm’ was defined as cough or phlegm for at least 3 consecutive months a year, for at least 2 years. ‘Chronic wheeze’ was defined as whistling sounds from chest (with or without cold), for at least 2 years. Shortness of breath was defined according to the Medical Research Council breathlessness scale which represent a spectrum of respiratory disability based on severity ranging from grade 1 to grade 5 [21]. Socio-economic status was defined using the proxy indicator of average monthly household income, which included income of all members living in the same house as well as additional earnings based on any business or other investment. ‘Ever smoker’ was defined as smoking more than 20 pack of cigarettes in lifetime or more than one cigarette a day for one year. ‘Pack years of smoking’ was defined as the number of cigarettes smoked per day divided by 20 and multiplied by the number of years that the person smoked. The variable ‘ethnicity’ was defined and categorized on the basis of five commonly spoken languages in Pakistan where minor variants and less commonly spoken dialects were merged with the more commonly spoken ones. For ‘educational level’ those who never attended school or did not know how to read or write were considered as illiterate while those who had been to school were categorized as literate. For the variable ‘type of cluster’; planned areas included those with permanent housing structure, sufficient living place, access to safe water and adequate sanitation system, while unplanned areas were densely populated areas of substandard housing, characterized by poverty, unsanitary and inferior living conditions and social disorganization [22]. ‘Animal or birds inside the house’ included both pets as well as animals kept as livestock. Type of kitchen was categorized based on its location, either inside or outside the house. Type of household was categorized based on construction of the household; *pakka* house refers to brick dwelling or concrete*, Kaccha-pakka* house refers to a mix of mud or thatched and brick dwelling. ‘Exposure to environmental tobacco smoke’ was defined as being exposed to cigarette smoke anywhere inside the house. Body mass index was defined according to WHO criteria for Asian population and categorized as: underweighted, < 18.5 kg/m^2^; normal, 18.5–23 kg/m^2^; overweight and obese, ≥ 23 kg/m^2^ [23]. ‘Current employment status’ was categorized as currently employed or self-employed and unemployed (included students, housewives, those currently not working anywhere or retired). The International Standard Classification of Occupations (ISCO) categories were grouped into three i.e. not working, high and low skilled blue collar workers (involved in manual work), high and low skilled white collar workers (involved in desk work) [24].

### Statistical methods

Data were entered twice and validated through Epi Data version 3.1 while analysis was done using SPSS version 19.0. Frequencies and proportions were calculated for categorical variables including the covariates listed above, as well as the respiratory symptoms and outcome variables based on the three definitions of asthma. Chi-square test was done to assess distribution of participants according to asthma outcome status. The age of the participant was categorized considering ten year interval from age 18 years and older; three categories were used where those aged 38 years or older were merged together. The variable pack-years of smoking was re-categorized to adjust for small cell count into three categories for multivariate analysis. Multi-collinearity was assessed using Eta, Phi and Cramer tests between covariates (cut off value > 0.5) and high collinearity was identified between gender and cooking status, as well as gender and occupational history. Interaction was checked between family history of asthma and history of allergies but it was not significant. The continuous variable of socio-economic status was categorized into tertiles and association was checked with outcome variables in univariate and multivariate models. Univariate logistic regression analysis was done to calculate unadjusted odds ratio of factors associated with two outcome variables; ‘reversibility in EFV_1_’ and ‘respiratory symptoms and reversibility in EFV_1_’. For the outcome defined as ‘self-reported asthma’, adjusted analysis could not be performed due to small proportion of asthmatics. The variables found to be significant in univariate models (*p*-value < 0.25) and those with biological plausibility were assessed further in the multivariate models. For covariates with more than two categories the cut-off of *p*-value < 0.25 was used for any one of the categories. Multivariate logistic regression was carried out to estimate the adjusted odds ratios for associated factors of asthma.

Ethical approval for the study was taken from Ethical Review Committee of Aga Khan University (2311-CHS-ERC-12). Prior to the interview, written informed consent was obtained from each respondent. The purpose and nature of the study was explained and spirometry procedure was demonstrated. Participants identified with any abnormality on spirometry were provided the report and were counselled and referred for further work-up and management.

## Results

Approximately 3000 adults aged 18 years and above were contacted to participate in the study in the selected households, out of which 1629 agreed to participate; giving a response rate of around 55%. Most of the non-responses were due to permanent relocation, or untraceable addresses and contact details. Participants who completed post-bronchodilator spirometry were 1054 and for those 930 spirograms were found to be acceptable according to standard guidelines. Majority (43%) of the participants belonged to the age group ≥38 years. Most common ethnicity among study participants was Urdu (44.6%) and majority of the participants were females (60%). Most of the study participants were currently unemployed (includes students, housewives, those currently not working anywhere or retired) (61%), while 20% were blue collar workers and 18.5% were white collar workers according to ISCO categories and 27% reported exposure to a dusty job. Ever smokers in our study were 13.5% while 28% reported exposure to environmental tobacco smoke. History of any allergy was reported by 28% while 12% reported positive family history of asthma (Table 1).

**Table 1:** Socio-demographic, anthropometric, household, lifestyle and occupational factors among adults ≥18 years, Karachi, Pakistan (*n* = 1629)

Characteristics	*n* (%)
Age	
18 to 27 years	531 (32.6)
28 to 37 years	399 (24.5)
≥ 38 years	699 (42.9)
Gender	
Male	658 (40.4)
Female	971 (59.6)
Birth Order	
1st	394 (24.2)
2nd	310 (19.0)
3rd	295 (18.1)
≥ 4th	630 (38.7)
Total number of children in household	
1 to 3	232 (14.3)
4 to 5	442 (27.1)
≥ 6	955 (58.6)
Ethnicity	
Urdu	715 (43.9)
Punjabi	469 (28.8)
Sindhi	295 (18.1)
Pushto	90 (5.5)
Baluchi	60 (3.7)
Educational level^a^ (*n* = 1626)	
Literate	1109 (68.2)
Illiterate	517 (31.8)
Socio-economic status^b^ (*n* = 1621)	
High-income	537 (33.0)
Middle-income	544 (33.4)
Low-income	540 (33.1)
Number of rooms in house	
1 room	293 (18.0)
≥ 2 rooms	1336 (82.0)
House ownership status	
Own	1223 (75.1)
Rented	407 (24.9)
Type of household	
*Pakka*	1579 (97.0)
*Kacha-Pakka*	50 (3.0)
Type of cluster^c^	
Planned	855 (52.5)
Unplanned	774 (47.5)
Wet spots inside house	844 (51.8)
Mold Inside house	81 (5.0)
Animal or birds inside house^d^	474 (29.0)
Carpeting inside house	528 (32.4)
Incense burning in house	767 (47.1)
Mosquito coil burning in house	739 (45.4)
Painted home in last 6 months	204 (12.5)
Cook food	894 (54.9)
Frequency of cooking food	
No cooking at all	735 (45.1)
Occasionally	143 (8.8)
Daily	751 (46.1)
Presence of window in kitchen	491 (30.1)
Presence of exhaust fan in kitchen	227 (13.9)
Type of kitchen	
Outdoor	632 (38.7)
Indoor separate	268 (16.5)
Indoor non-separate	729 (44.8)
Smoking status^e^	
Never	1409 (86.5)
Ever	220 (13.5)
Pack years of smoking^f^	
Non smoker	1409 (86.5)
≤ 10	132 (8.1)
10–20	31 (1.9)
> 20	57 (3.5)
Exposure to environmental tobacco smoke^g^	452 (28.1)
Body Mass Index^h^ (*n* = 1611)	
Underweight	673 (41.8)
Normal weight	575 (35.7)
Overweight and obese	363 (22.5)
History of any allergy	451 (27.7)
Family history of asthma	192 (11.8)
Family History of tuberculosis	44 (2.7)
Exposure to any dusty job	
Never worked	899 (55.2)
Working and no dust exposure	293 (18.0)
Working and dust exposure	437 (26.8)
Exposure to gas or fumes at work	
Never worked	899 (55.2)
Working and no gas exposure	592 (36.3)
Working and gas exposure	138 (8.5)
Current employment status^i^	
Unemployed	1000 (61.4)
Employed	629 (38.6)
ISCO Categories^j^	
Not working	1000 (61.4)
White collar worker	301 (18.5)
Blue collar worker	328 (20.1)

^a^Educational level: those who never attended school or did not know how to read or write were considered as illiterate while those who had been to school were categorized as literate

^b^Socio-economic status was defined using the proxy indicator of monthly household income which included income of all members living in the same house as well as additional earnings based on any business or other investment

^c^Type of cluster was defined as planned areas included those with permanent housing structure, sufficient living place, access to safe water and adequate sanitation system, while unplanned areas were densely populated areas of substandard housing, characterized by poverty, unsanitary and inferior living conditions and social disorganization

^d^Animal or birds inside house included both pets as well as animals kept as livestock

^e^Ever smoker was defined as smoking more than 20 packs of cigarettes in a lifetime or more than one cigarette a day for one year

^f^Pack years of smoking was defined as the number of cigarettes smoked per day divided by 20 and multiplied by the number of years that the person smoked

^g^Exposure to environmental tobacco smoke was defined as anyone who smoked cigarettes anywhere inside the house

^h^Body mass index was defined according to WHO criteria for Asian population and categorized as: underweighted, < 18.5 kg/m^2^; normal, 18.5–23 kg/m2; overweight and obese, ≥ 23 kg/m^2^

^i^Current employment status was defined as employed somewhere currently or self-employed, whereas, unemployed included students, housewives, those currently not working anywhere or retired

^j^The International Standard Classification of Occupations (ISCO) categories were three i.e. not working, high and low skilled blue collar workers (involved in manual work), high and low skilled white collar workers (involved in desk work)

The prevalence of self-reported asthma was 1.8% (95% CI: 1.0–2.6), while prevalence according to reversibility in FEV_1_ was 11.3% (95% CI: 9.4–13.3) and symptoms and reversibility in FEV_1_ was 6.6% (95% CI: 5.1–8.1) (Table 2). The break-up by sex (female/male) was: self-reported asthma: 1.6% /2.0%, reversibility in FEV_1_: 10.8% /11.7% and symptoms and reversibility in FEV_1_: 6.9% /6.2%.

**Table 2:** Prevalence of asthma and respiratory symptoms among adults ≥18 years, Karachi, Pakistan, 2015 (*n* = 1629)

Outcome variables	*n* (%)	95% CI
Self-reported asthma^a^	29 (1.8)	1.0–2.6
Reversibility ≥200 ml in FEV_1_^b d^	105 (11.3)	9.4–13.3
Respiratory symptoms and reversibility ≥200 ml in FEV_1_^C d^	61 (6.6)	5.1–8.1
SOB grade I	410 (25.2)	22.5–27.9
SOB grade II	359 (22.0)	19.5–24.6
Acute cough	72 (4.4)	3.1–5.7
Chronic cough	49 (3.0)	1.9–4.1
Acute wheeze	164 (10.1)	8.2–11.9
Chronic wheeze	130 (8.0)	6.3–9.7
Acute phlegm	107 (6.6)	5.1–8.1
Chronic phlegm	60 (3.7)	2.5–4.9

^d^Sample size: 930 participants

*SOB* Shortness of breath

^a^Self-reported, physician-diagnosed asthma

^b^Spirometry-based asthma assessed on presence of post-bronchodilator reversibility ≥200 ml in FEV_1_

^c^Asthma based on post-bronchodilator reversibility ≥200 ml in FEV_1_ and one or more respiratory symptoms, or self-reported asthma

Among those with self-reported asthma (*n* = 29), common triggering factors for aggravation of asthma included; exposure to smoke (83%), strong smell (53%) and dust (83%). Participants currently on prescribed medications for asthma were 80% and out of these 71% participants were taking inhaled bronchodilators whereas 42% were on oral medications.

The prevalence of respiratory symptoms identified in this study was: acute cough, 4.4% (95% CI: 3.1–5.7); acute wheeze, 10.1% (95% CI: 8.2–11.9); and shortness of breath (Grade I), 25.2% (95% CI: 22.5–27.9) (Table 2).

Various factors found to be associated with self-reported asthma include: age ≥ 38 years; presence of two or more rooms in house; living in a *Kacha-Pakka* house; pack years of smoking for ≥20 years; history of any allergy and family history of asthma (see Additional file 1).

Univariate logistic regression analysis found that asthmatics based on reversibility in FEV_1_ were more likely to belong to the age group ≥38 years (OR 1.9, 95% CI: 1.1 to 3.2), low socio-economic status (OR 1.8, 95% CI: 1.1 to 3.1), were ever smokers (OR 1.6, 95% CI: 1.1 to 2.6), and those with pack years of smoking of 5 years or more (OR 2.2, 95% CI: 1.2 to 4.1) and those with history of any allergy (OR 1.7, 95% CI: 1.1 to 2.6). Asthmatics according to respiratory symptoms and reversibility in FEV_1_ were more likely to belong to the age group ≥38 years (OR 2.2, 95% CI: 1.1 to 4.2), were illiterate (OR 1.9, 95% CI: 1.1 to 3.2), were among those with pack years of smoking of 5 years or more (OR 3.2, 95% CI: 1.7 to 6.3), had exposure to environmental tobacco smoke (OR 1.8, 95% CI: 1.1 to 3.1), had history of allergies (OR 2.7, 95% CI: 1.6 to 4.5) and had family history of asthma (OR 2.0, 95% CI: 1.1 to 3.9) (Table 3).

**Table 3:** Univariate logistic regression analysis for factors associated with asthma among adults ≥18 years, Karachi, Pakistan (*n* = 930)

Characteristics	Reversibility in FEV_1_^k^		Respiratory symptoms and reversibility in FEV_1_^k^	
	OR (95% CI)	*P*- Value	OR (95% CI)	*p*-value
Age				
18 to 27 years	1	0.02	1	0.01
28 to 37 years	0.9 (0.5–1.8)	0.51	0.7 (0.3–1.8)	0.47
≥ 38 years	1.9 (1.1–3.2)	0.03	2.2 (1.1–4.2)	0.02
Gender				
Male	1		1	
Female	0.9 (0.6–1.4)	0.40	1.1 (0.7–1.9)	0.64
Birth Order				
1st	0.8 (0.5–1.5)	0.51	0.8 (0.4–1.6)	0.48
2nd	0.9 (0.6–1.7)	0.61	0.8 (0.4–1.8)	0.67
3rd	1.0 (0.6–1.8)	0.31	1.4 (0.7–2.7)	0.37
≥ 4th	1		1	
Total number of Children				
1 to 3	0.9 (0.5–1.6)	0.22	0.9 (0.5–1.7)	0.21
4 to 5	0.9 (0.5–1.4)	0.81	0.5 (0.2–1.4)	0.82
≥ 6	1		1	0.44
Education^a^				
Literate	1		1	
Illiterate	1.4 (0.9–2.1)	0.05	1.9 (1.1–3.2)	0.02
Socio-economic status^b^				
High income	1	0.21	1	0.13
Middle income	1.6 (0.9–2.6)	0.10	0.9 (0.5–1.7)	0.22
Low income	1.8(1.1–3.1)	0.03	1.2 (0.6–2.2)	0.24
Number of rooms				
≥ 2 rooms	1		1	
1 room	1.1 (0.7–1.8)	0.75	1.1 (0.6–2.2)	0.71
Ownership status				
Own	1		1	
Rented	0.9 (0.6–1.5)	0.31	1.3 (0.7–2.2)	0.44
Type of cluster^c^				
Planned	1		1	
Unplanned	1.0 (0.7–1.6)	0.29	0.9 (0.5–1.5)	0.73
Wet spots inside house				
No	1		1	
Yes	1.1 (0.7–1.6)	0.28	0.9 (0.6–1.7)	0.99
Animal or birds inside house^d^				
No	1		1	
Yes	0.7 (0.4–1.2)	0.19	0.6 (0.3–1.1)	0.10
Carpeting inside house				
No	1		1	
Yes	0.9 (0.6–1.4)	0.81	0.9 (0.6–1.7)	0.88
Incense burning in home				
Never	1		1	
Ever	1.0 (0.7–1.5)	0.96	1.0 (0.6–1.7)	0.97
Mosquito coil				
Never	1		1	
Ever	0.7 (0.5–1.1)	0.85	0.9 (0.6–1.6)	0.91
Cooking food				
No	1		1	
Yes	1.0 (0.7–1.5)	0.41	0.5 (0.3–1.1)	0.47
Window in Kitchen				
Yes	1		1	
No	0.9 (0.6–1.3)	0.08	0.6 (0.4–1.0)	0.05
Exhaust in kitchen				
Yes	1		1	
No	0.8 (0.5–1.5)	0.51	0.5 (0.3–0.9)	0.04
Type of kitchen				
Outdoor	1		1	0.401
Indoor separate	1.5 (0.8–2.6)	0.15	1.6 (0.8–3.4)	0.17
Indoor non separate	1.2 (0.7–1.9)	0.21	1.3 (0.7–2.4)	0.41
Smoking status^e^				
Never	1		1	
Ever	1.6 (1.1–2.6)	0.03	1.7 (0.9–3.1)	0.07
Pack years of smoking^f^				
Non smoker	1	0.00	1	0.001
< 5 years	1.1 (0.5–2.4)	0.39	0.6 (0.2–2.1)	0.47
≥ 5 years	2.2 (1.2–4.1)	0.001	3.2 (1.7–6.3)	0.001
Exposure to environmental tobacco smoke^g^				
No	1		1	
Yes	1.5 (0.9–2.3)	0.05	1.8 (1.1–3.1)	0.03
Body Mass Index^h^				
Underweight	1	0.490	1	0.410
Normal	1.4 (0.9–2.2)	0.100	1.4 (0.8–2.6)	0.287
Overweight and obese	1.3 (0.8–2.3)	0.101	1.6 (0.8–3.1)	0.208
History of any allergy				
No	1		1	
Yes	1.7 (1.1–2.6)	0.02	2.7 (1.6–4.5)	0.001
Family history of asthma				
No	1		1	
Yes	1.0 (0.5–1.9)	0.41	2.0 (1.1–3.9)	0.00
Exposure of dusty job				
Not working	1	0.81	1	0.82
Working and no dust exposure	0.9 (0.5–1.5)	0.42	0.4 (0.1–0.9)	0.04
Working and dust exposure	1.4 (0.9–2.1)	0.39	1.2 (0.7–2.2)	0.42
Exposure of gas or fumes at work				
Not working	1	0.10	1	0.91
Working and no gas exposure	1.1 (0.7–1.7)	0.41	0.9 (0.5–1.6)	0.68
Working and gas exposure	1.5 (0.8–2.8)	0.35	0.9 (0.4–2.3)	0.97
Current employment status^i^				
Unemployed	1		1	
Employed	1.1 (0.7–1.7)	0.51	0.9 (0.5–1.5)	0.701
ISCO Categories ^j^				
Not working	1	0.78	1	0.88
White collar worker	0.9 (0.5–1.5)	0.59	0.8 (0.4–1.7)	0.62
Blue collar worker	1.3 (0.8–2.1)	0.34	0.95 (0.5–1.8)	0.87

^a^Educational level: those who never attended school or did not know how to read or write were considered as illiterate while those who had been to school were categorized as literate

^b^Socio-economic status was defined using the proxy indicator of monthly household income which included income of all members living in the same house as well as additional earnings based on any business or other investment

^c^Type of cluster was defined as planned areas included those with permanent housing structure, sufficient living place, access to safe water and adequate sanitation system, while unplanned areas were densely populated areas of substandard housing, characterized by poverty, unsanitary and inferior living conditions and social disorganization

^d^Animal or birds inside house included both pets as well as animals kept as livestock

^e^Ever smoker was defined as smoking more than 20 packs of cigarettes in a lifetime or more than one cigarette a day for one year

^f^Pack years of smoking was defined as the number of cigarettes smoked per day divided by 20 and multiplied by the number of years that the person smoked

^g^Exposure to environmental tobacco smoke was defined as anyone who smoked cigarettes anywhere inside the house

^h^Body mass index was defined according to WHO criteria for Asian population and categorized as: underweighted, < 18.5 kg/m2; normal, 18.5–23 kg/m^2^; overweight and obese, ≥ 23 kg/m^2^

^i^Current employment status was defined as employed somewhere currently or self-employed, whereas, unemployed included students, housewives, those currently not working anywhere or retired

^j^The International Standard Classification of Occupations (ISCO) categories were three i.e. not working, high and low skilled blue collar workers (involved in manual work), high and low skilled white collar workers (involved in desk work)

^k^Definitions used for asthma:

Spirometry-based asthma assessed on presence of post-bronchodilator reversibility ≥200 ml in FEV1

Asthma based on post-bronchodilator reversibility ≥200 ml in FEV1 and one or more respiratory symptoms, or self-reported asthma

Multivariate logistic regression models found that participants categorized as asthmatics on reversibility in FEV_1_ were more likely to belong to the age group ≥38 years (AOR 1.9, 95% CI: 1.2 to 3.3), low socioeconomic group (AOR 1.9, 95% CI: 1.2 to 3.4), to have history of allergies (AOR 1.9, 95% CI: 1.2 to 2.9), exposure to environmental tobacco smoke (AOR 1.6, 95% CI: 1.1 to 2.5). While asthmatics based on respiratory symptoms and reversibility in FEV_1_ were more likely belong to age group ≥38 years (AOR 2.1, 95% CI: 1.1 to 4.2), reported 5 or more pack years of smoking (AOR 2.3, 95% CI: 1.1 to 4.7), reported exposure to environmental tobacco smoke (AOR 1.9, 95% CI: 1.1 to 3.3) and had history of any allergy (AOR 2.8, 95% CI: 1.7 to 4.8) (Table 4).

**Table 4:** Multivariate logistic regression analysis for factors associated with asthma among adults ≥18 years, Karachi, Pakistan (*n* = 930)

Characteristics	Reversibility ≥200 ml in FEV_1_^k^	Respiratory symptoms and reversibility in FEV_1_^k^
	AOR (95% CI)	AOR (95% CI)
Age		
18 to 27 years	1	1
28 to 37 years	0.9 (0.5–1.7)	0.7 (0.3–1.8)
≥ 38 years	1.9 (1.2–3.3)	2.1 (1.1–4.2)
Socio-economic status^a^		
High income	1	–
Middle income	1.7 (0.9–2.8)	–
Low income	1.9 (1.2–3.4)	–
History of any allergy		
No	1	1
Yes	1.9 (1.2–2.9)	2.8 (1.7–4.8)
Pack years of smoking^b^		
Non smoker	–	1
< 5 years	–	0.6 (0.2–1.9)
≥ 5 years	–	2.3 (1.1–4.7)
Exposure to environmental tobacco smoke^c^		
No	1	1
Yes	1.6 (1.1–2.5)	1.9 (1.1–3.3)

^a^Socio-economic status was defined using the proxy indicator of monthly household income which included income of all members living in the same house as well as additional earnings based on any business or other investment

^b^Pack years of smoking was defined as the number of cigarettes smoked per day divided by 20 and multiplied by the number of years that the person smoked

^c^Exposure to environmental tobacco smoke was defined as anyone who smoked cigarettes anywhere inside the house

^k^Definitions used for asthma:

Spirometry-based asthma assessed on presence of post-bronchodilator reversibility ≥200 ml in FEV_1_

Asthma based on post-bronchodilator reversibility ≥200 ml in FEV_1_ and one or more respiratory symptoms, or self-reported asthma

## Discussion

To the best of our knowledge, this is the first community-based epidemiological assessment identifying prevalence of adult asthma in Pakistan. Globally, there is a lack of reliable estimates for asthma and a lack of agreement on standardized criteria for epidemiological assessment of asthma and therefore we believe that this study fills an important gap in knowledge by providing reliable estimates on adult asthma using a combination of respiratory symptoms and objective assessment. This study found significant unreported asthma with the use of spirometry in the city of Karachi, Pakistan; 11.3 and 6.6% for ‘reversibility in FEV_1_’ and ‘respiratory symptoms and reversibility in FEV_1_’ respectively; while reporting comparatively lower prevalence (1.8%) for self-reported asthma.

Prevalence of self-reported asthma (1.8%) in this study was comparable to regional estimates; approximately 2% in India and Thailand [7, 14]. In the Indian INSEARCH survey, self-reported asthma was diagnosed based on asthma symptoms, previous diagnosis of asthma or medication use [7]. Whereas, the survey from Thailand also identified asthma based on self-reported physician diagnosed asthma accompanied by any asthma symptoms or use of medications [14]. However, our study estimate was lower than that reported by a national survey in Iran, using symptoms of wheezing with dyspnea, as 9.4%; and also somewhat lower when using their definition of ‘current asthma’ which included asthma attack or use of asthma medications i.e. 4.7% [8]. Obel et al. reported asthma burden to be 6.9% in a population based survey from sub-Saharan Africa using self-reported asthma-ever and and/or receiving asthma medications, which is higher than in our study [25]. Furthermore, South Asian women including those of Pakistani origin living in the United Kingdom were found to have a self-reported asthma prevalence of 10.9% [15]. Some of these differences are due to the use of varying definitions for asthma while the study from the UK may reflect an additional risk of asthma among migrants from South Asian countries. The use of the variable physician-diagnosed asthma may also be unreliable due to the varying capacity of general practitioners to identify asthma in different countries [6].

Prevalence of asthma based on reversibility in FEV_1_ was 11.3% in this study, which is comparable to prevalence reported from Iran of 9.4% [26]. We believe that our study identified a previously unreported burden of asthma through spirometry. Though this is a robust and objective assessment, spirometry may still underestimate the actual prevalence of asthma due to the less sensitive nature of the reversibility test as compared to physicians’ assessment [27]. There is a probability of people who fail to accomplish the reversibility criteria significantly on a single occasion but who actually have asthma [27].

Lower prevalence rates of 2.9 and 2.3% have been reported from Thailand and South Australia [14, 27], respectively. Whereas, prevalence found in our study was 6.6%, comparatively higher based on respiratory symptoms and reversibility in FEV_1_. The study by Dejsomritrutai et al. among Thai adults was a community based survey which identified asthma on reversibility in spirometry or having any respiratory symptoms, in addition to bronchial hyper-responsiveness on broncho-provocation test [14]. Furthermore, Amiri et al. reported 9% prevalence from a cross-sectional survey from Iran where asthma assessed through spirometry [26]. De Marco et al. reported that asthma prevalence may be overestimated if assessed through self-reported wheezing but might be underestimated if assessed through self-reported asthma and clinical judgment [28]. Our study also supports the use of a combination of lung function and respiratory symptoms data for assessing asthma and identified a wide gap between self-reported asthma and substantial unidentified burden of asthma in the community.

Prevalence of acute and chronic wheeze reported in this study was 10 and 8% respectively, while prevalence of wheeze in past 12 months reported in a study from India was 2.6% [7]. Prevalence for shortness of breath (Grade I) found in this study was 25%, while prevalence of breathlessness on exertion was reported by 5% in India, which might be due to over-reporting in our study [7].

The prevalence of acute and chronic cough reported in our study was 4.4 and 3%, respectively, which is comparable to the prevalence of chronic cough found in India, i.e. 4.6% [7]. Prevalence of acute and chronic phlegm was found to be 6.6 and 3.7% respectively in this study which is also similar to 3.8% prevalence of chronic phlegm reported from India [7].

A study conducted in Mashhad, Iran, found that history of allergy was an important risk factor for asthma, a finding which is similar to our study which found history of allergy to be associated with asthma according to both definitions ‘reversibility in FEV_1_’ and ‘respiratory symptoms and reversibility in FEV_1_’ with odds ratio of AOR (95% CI): 1.9 (1.2–2.9) and 2.8 (1.7–4.8) respectively. [29]. Allergy has been commonly reported risk factor across many studies and established associations have also been found for high Serum IgE level, a substitute for allergic sensitization, and for common allergens identified by skin prick tests with asthma [3, 30]. A nationally representative survey among Iranian adults showed that prevalence of asthma was significantly higher among the older age group [8]. This finding is comparable to the INSEARCH survey from India which determined significant relationship with advancing age, highlighting a possible “cumulative effect” of age, including asthma diagnosed in past, besides late onset of the disease [7]. New onset asthma may also occur in adulthood because of prolonged environmental or occupational exposures to respiratory irritants [3]. High asthma prevalence has been observed progressively with increasing age in Asia [5]. Likewise, evidence suggests that older people with a history of smoking may develop asthma-COPD overlap syndrome (ACOS) [31]. People with COPD might have airway responsiveness, have shown reversibility in FEV_1_ and therefore may be categorized as ACOS. Such cases may have overestimated the prevalence in our study however, symptoms suggestive of asthma were also taken into account in one of the definitions in order to overcome such over-reporting [32].

Tobacco smoking is known to cause inflammatory changes of the airway tract, resulting in developing or worsening of pre-existing asthma symptoms; however, there is inconsistency of evidence regarding association of tobacco smoking and adult asthma [2, 33]. An Indian survey found smoking to be an important risk factor for asthma [11]. Further, Swedish studies reported population attributable risk for asthma symptoms attributed to smoking between 9.8 to 25.5% [33]. Recently, a follow up study suggested that smokers with asthma tend to have more complaint of chronic cough and phlegm as compared to non-smokers [34]. These findings are consistent with our study where we found an association between tobacco smoking and asthma according to “respiratory symptoms and reversibility in FEV_1_” AOR (95% CI): 2.3 (1.1–4.7).

Previous studies found that environmental tobacco smoke exposure, one of the consistent modifiable risk factor, played a strong role in developing and aggravating asthma [3, 35]. Our finding for environmental tobacco smoke AOR (95% CI): 1.6 (1.1–2.5) and 1.9 (1.1–3.3) respectively for definitions based on ‘reversibility in FEV_1_’ and ‘respiratory symptoms and reversibility in FEV_1_’ was consistent with other studies [3]. European Community Respiratory Health Survey (ECRHS) findings suggest that maternal smoking during pregnancy or post-natal period as well as paternal smoking is associated with development of symptoms suggestive of asthma and had detrimental effect on lung function of children later in the adulthood [36]. Socioeconomic position has an important role to be identified for preventing inequalities and overall decreasing disease burden [37]. In our study, significant association of lower socioeconomic status has been found with asthma AOR (95% CI): 1.9 (1.2–2.9). This finding was consistent with a multicenter study which found low socio-economic position as a consistent risk factor for asthma [37]. Another cohort study reported association of lower socioeconomic position with the asthma onset in adulthood [38]. Our study could not find any significant association of asthma with gender, which is similar to findings of a population based study from Iran [8]. There is some inconsistent evidence suggesting possible role of hormonal changes during puberty for greater likelihood of asthma among women [39].

Some limitations need to be considered for this study. We had a relatively high non-response rate of 45% which may be attributed to a generally poor security situation in the city at the time of data collection, resulting in limited access for study teams. However, spirometry based surveys have been found to report low response rates, such as studies conducted in Thailand, Tanzania and Sweden [14, 40, 41]. Probable reasons for low participation in spirometry based surveys may include apprehensions for the spirometry procedure, which requires physical exertion. Since the study was conducted in urban setting, hence, generalizability may be limited to similar urban population in Pakistan. However, we believe that the population in Karachi represents common ethnic groups of the country; therefore our findings are applicable for various ethno-linguistic groups from urban areas of the country.

There are several strengths which need to be considered for this study as well. This was the first community based respiratory health survey conducted in Pakistan employing robust methodology and objective lung function assessment. This study used standard ATS guidelines and protocols for conducting spirometry for asthma assessment, along with WHO Global Health Survey based standard definitions of physician-diagnosed asthma [19, 20]. American Thoracic Society respiratory questionnaire (ATS-DLD-78A) was used in this study for assessment of respiratory symptoms, which has been validated both internationally and locally in Pakistan [17]. In addition, a sampling frame was developed prior to data collection for purpose of random selection of households which adds to the strengths of this study. We believe that this study would have a substantial research impact in terms of providing much needed empirical evidence related to respiratory epidemiology on prevalence of asthma in Pakistan via three different definitions. The information obtained from this study may be utilized for priority-setting and developing strategic public health plans for the optimal resource allocation for preventive and curative programs.

## Conclusion

This study provides a robust community based epidemiological assessment for actual prevalence of adult asthma in Pakistan, reporting a high prevalence in the country. The study identified age ≥ 38 years, low socio-economic status, history of allergies, exposure to environmental tobacco smoke and ≥ 5 pack-years of smoking as important risk factors for adult asthma in the country. Our findings have strong implications for incorporating chronic respiratory diseases, including asthma, among priority non-communicable diseases in the national and regional health policies of the country, with adequate provision for both curative and preventive strategies.

## Additional file

Additional file 1: **Table S1.** Frequency distribution of socio-demographic, anthropometric, household, lifestyle and occupational factors among adults ≥18 years according to self-reported physician-diagnosed asthma, Karachi, Pakistan (*n* = 1629). (DOCX 16 kb)

## Abbreviations

AOR: Adjusted odds ratio
CI: Confidence interval
FEV_1_: Forced expiratory volume in first second
FVC: Forced Vital Capacity
