# Supplementary material for: Epidemiology of asthma and associated factors in an urban Pakistani population: adult asthma study-Karachi
Source: BMC Pulm Med. 2018 Dec 4;18:184. doi: 10.1186/s12890-018-0753-y (PMC6278017; doi:10.1186/s12890-018-0753-y)
Supplement: Supplementary file 1 — Table S1. Frequency distribution of socio-demographic, anthropometric, household, lifestyle and occupational factors among adults ≥18 years according to self-reported physician-diagnosed asthma, Karachi, Pakistan (n = 1629). (DOCX 16 kb) [file 12890_2018_753_MOESM1_ESM.docx]

Table S1: Frequency distribution of socio-demographic, anthropometric, household, lifestyle and occupational factors among adults ≥ 18 years according to self-reported physician-diagnosed asthma, Karachi, Pakistan (n=1629)

| Characteristics | Asthma n (%)  n=29 | No Asthma n (%)  n=1600 |
| --- | --- | --- |
| Age |  |  |
| 18 to 27 years | 6 (20.7) | 525 (32.8) |
| 28 to 37 years | 1 (3.4) | 398 (24.9) |
| ≥ 38 years | 22 (75.9) | 677 (42.3) |
| Gender |  |  |
| Male | 13 (44.8) | 645 (40.3) |
| Female | 16 (55.2) | 955 (59.7) |
| Birth Order |  |  |
| 1^st^ | 11 (37.9) | 383 (23.9) |
| 2^nd^ | 7 (24.1) | 303 (18.9) |
| 3^rd^ | 6 (20.7) | 289 (18.1) |
| ≥ 4^th^ | 5 (17.2) | 625 (39.1) |
| Total number of Children † |  |  |
| 1 to 3 | 2 (6.9) | 230 (14.4) |
| 4 to 5 | 12 (41.4) | 430 (26.9) |
| ≥ 6 | 15 (51.7) | 940 (58.7) |
| Ethnicity † |  |  |
| Urdu | 16 (55.2) | 699 (43.7) |
| Punjabi | 5 (17.2) | 464 (29.0) |
| Sindhi | 7 (24.1) | 288 (18.0) |
| Pushto | 1 (3.4) | 89 (5.6) |
| Baluchi | 0 | 60 (3.8) |
| Education ^a^ (n=1626) |  |  |
| Literate | 18 (62.1) | 1091 (68.3) |
| Illiterate | 11 (37.9) | 506 (31.7) |
| Socio-economic status ^b^ (n=1621) |  |  |
| High income | 10 (34.5) | 527 (33.1) |
| Middle income | 5 (17.2) | 539 (33.9) |
| Low income | 14 (48.3) | 526 (33.0) |
| Number of rooms * |  |  |
| 1 room | 1 (3.4) | 292 (18.3) |
| ≥ 2 rooms | 28 (96.6) | 1308 (81.8) |
| Ownership status |  |  |
| Own | 21 (72.4) | 1201 (75.1) |
| Rented | 8 (27.6) | 399 (24.9) |
| Type of household† * |  |  |
| Pakka | 26 (89.7) | 1553 (97.1) |
| Kacha Pakka | 3 (10.3) | 47 (2.9) |
| Type of cluster ^c^ |  |  |
| Planned | 16 (55.2) | 839 (52.4) |
| Unplanned | 13 (44.8) | 761 (47.6) |
| Wet spots inside house | 13 (44.8) | 831 (51.9) |
| Mold Inside house † | 2 (6.9) | 79 (4.9) |
| Animal or birds inside house ^d^ | 07 (24.1) | 467 (29.2) |
| Carpeting inside house | 8 (27.6) | 520 (32.5) |
| Incense burning inside home | 14 (48.3) | 753 (47.1) |
| Mosquito coil burning inside house | 11 (37.9) | 728 (45.5) |
| Painted home in last six months † | 4 (13.8) | 200 (12.5) |
| Cook food | 13 (44.8) | 881 (55.1) |
| Frequency of Cooking Food † |  |  |
| No cooking at all | 16 (55.2) | 719 (44.9) |
| Occasionally | 2 (6.9) | 141 (8.8) |
| Daily | 11 (37.9) | 740 (46.3) |
| Window in Kitchen | 12 (41.4) | 479 (29.9) |
| Exhaust in Kitchen † | 5 (17.2) | 222 (13.9) |
| Type of kitchen † |  |  |
| Outdoor | 11 (37.9) | 621 (38.8) |
| Indoor separate | 7 (24.1) | 261 (16.3) |
| Indoor non separate | 11 (37.9) | 718 (44.9) |
| Smoking status ^e^ † * |  |  |
| Never | 21 (72.4) | 1388 (86.8) |
| Ever | 8 (27.6) | 212 (13.2) |
| Pack years of smoking ^f^ † * |  |  |
| Non smoker | 21 (72.4) | 1388 (86.8) |
| 0 years till 10 years | 01 (3.4) | 131 (8.2) |
| >10 years till 20 years | 01 (3.4) | 30 (1.9) |
| >20 years | 06 (20.7) | 51 (3.2) |
| Exposure to environmental tobacco smoke ^g^ | 8 (27.6) | 444 (27.8) |
| Body Mass Index ^h^ (n=1611) |  |  |
| Underweight | 12 (41.4) | 661 (41.8) |
| Normal | 13 (44.8) | 562 (35.5) |
| Overweight and obese | 4 (13.8) | 359 (22.7) |
| History of any allergy * | 23 (79.3) | 428 (26.8) |
| Family history of asthma ^†^ * | 15 (51.7) | 177 (11.1) |
| Family History of TB ^†^ | 0 | 44 (2.8) |
| Exposure of dusty job |  |  |
| Never worked | 17 (58.6) | 882 (55.1) |
| Working and no dust exposure | 3 (10.3) | 290(18.1) |
| Working and dust exposure | 9 (31.0) | 428 (26.8) |
| Exposure of gas or fumes at work ^†^ |  |  |
| Never worked | 17 (58.6) | 882 (55.1) |
| Working and no gas exposure | 9 (31.0) | 583 (36.4) |
| Working and gas exposure | 3 (10.3) | 135 (8.4) |
| Current employment status ^i^ |  |  |
| Unemployed | 19 (65.5) | 981 (61.3) |
| Employed | 10 (34.5) | 619 (38.7) |
| ISCO Categories ^j^ |  |  |
| Not working | 19 (65.5) | 981 (61.3) |
| White collar worker | 4 (13.8) | 297 (18.6) |
| Blue collar worker | 6 (20.7) | 322 (20.1) |

^†^Expected cell count < 5; Fisher’s exact test was performed

*Significant at *p* < 0.05

^a^ Educational level: those who never attended school or did not know how to read or write were considered as illiterate while those who had been to school were categorized as literate

^b^ Socio-economic status: was defined using the proxy indicator of monthly household income which included income of all members living in the same house as well as additional earnings based on any business or other investment.

^c^ Type of cluster was defined as planned areas included those with permanent housing structure, sufficient living place, access to safe water and adequate sanitation system, while unplanned areas were densely populated areas of substandard housing, characterized by poverty, unsanitary and inferior living conditions and social disorganization

^d^ Animal or birds inside house included both pets as well as animals kept as livestock

^e^ Ever smoker was defined as smoking more than 20 packs of cigarettes in a lifetime or more than one cigarette a day for one year

^f^ Pack years of smoking was defined as the number of cigarettes smoked per day divided by 20 and multiplied by the number of years that the person smoked

^g^ Exposure to environmental tobacco smoke was defined as anyone who smoked cigarettes anywhere inside the house

^h^ Body mass index was defined according to WHO criteria for Asian population and categorized as: underweighted, <18.5 kg/m2; normal, 18.5-23 kg/m2; overweight and obese, ≥ 23 kg/m2

^i^ Current employment status was defined as employed somewhere currently or self-employed, whereas, unemployed included students, housewives, those currently not working anywhere or retired

^j^ The International Standard Classification of Occupations (ISCO) categories were three i.e. not working, high and low skilled blue collar workers (involved in manual work), high and low skilled white collar workers (involved in desk work)
